# Supplementary material for: Pattern mining and prediction techniques for user behavioral trajectories in e-commerce
Source: PLoS One. 2025 May 16;20(5):e0320772. doi: 10.1371/journal.pone.0320772 (PMC12083830; doi:10.1371/journal.pone.0320772)
Supplement: Attachment — Submitted filename: renamed_95795.doc [file pone.0320772.s003.doc]

**Detailed Response to Reviewers**

**Title: Pattern Mining and Prediction Methods for User Behavior Trajectories in**

**E-Commerce**

**Journal title: Plos ONE**

**Dear Editor and Dear reviewers:**

Thank you for your useful comments on our manuscript. We wish to give a sincere

gratitude to anonymous referees for reviewing our paper carefully. We apologize

for any inconveniences caused by these errors. We have modiﬁed the manuscript

accordingly, and the speciﬁc modiﬁcations are listed point by point below.

**Reviewer: 1**

1. It is an excellent study however, the expression is weak. The structure of the article is incoherent and needs critical review by the authors. The headings and subheadings needs to be revisited along with grammatical errors such as tenses used in a single paragraph are incoherent. Clearly break down the draft into literature and methodological portions. A few aspects have been highlighted on the attached manuscript.

**Thank you for your feedback. We will enhance the expression, review the structure for coherence, correct grammatical inconsistencies, and separate the draft into clear literature and methodology sections. We will also address the highlighted aspects in the manuscript.**

**Reviewer: 2**

1. I wanted to take a moment to compliment you on your outstanding eﬀort with this paper. The clarity with which you express complex concepts is admirable, and your meticulous analysis contributes signiﬁcantly to the subject. A well-written paper, however, it is recommended to add limitations and future directions of your study.

**Thank you for your kind words and appreciation. I’m glad the paper resonated with you. I will deﬁnitely consider adding sections on limitations and future directions to provide a more comprehensive view of the study. Your suggestions are valuable, and I appreciate your input. .**

**Reviewer: 3**

1. The article “Pattern Mining and Prediction Techniques for User Behavioral Trajectories in E-Commerce” is very interesting. It was a pleasure to evaluate it.

This article presents a comprehensive analysis of pattern mining and prediction techniques for user behavioral trajectories in the context of e-commerce. The authors address relevant issues related to the analysis of user behavior sequences, proposing new methods to improve the accuracy of predictions and the eﬀectiveness of data analysis. As strengths, the study addresses a topic of importance in the current e-commerce scenario, oﬀering insights for researchers and professionals in the ﬁeld. The article provides a thorough explanation of the proposed methods, facilitating the understanding of the study. The inclusion of practical experiments strengthens the study’s conclusions and demonstrates the applicability of the proposed techniques. Regarding areas for improvement, in the theoretical foundation, the initial sections, especially the ﬁrst four paragraphs, lack references. It is recommended to include more citations to contextualize the work in the existing literature. The section addressing the study’s limitations (page 11) could be expanded, including comparisons with previous works and discussing possible solutions for the identiﬁed limitations. Sections 2, 3, 4, 5, and 6 would beneﬁt from a revision to include more bibliographic references, strengthening the theoretical basis of the work. An interesting point to include would be a debate on whether there are ethical considerations related to user data privacy that should be discussed, and the scalability of the proposed methods for larger datasets. My recommendations for adjustments would be: Include a more comprehensive review of existing literature, especially in the initial sections and in the discussion of limitations, and expand the discussion on the practical and ethical implications of the results for e-commerce professionals.

The article presents a signiﬁcant contribution to the ﬁeld of user behavior analysis in e-commerce. With the suggested improvements, especially regarding the theoretical foundation and discussion of limitations, the work has the potential to be a high-impact publication in the area.

**Thank you for your insightful evaluation of the article “Pattern Mining and Prediction Techniques for User Behavioral Trajectories in E-Commerce.” We appreciate your recognition of the study’s strengths and the value it brings to the ﬁeld. We deﬁnitely consider your suggestions to enhance the theoretical foundation by adding more references, particularly in the initial sections. We have added additional references, speciﬁcally numbers 5-11, 29-30, and 37-50.We also give some limitations discussion. We also agree on the importance of discussing ethical considerations. Your recommendations for a more comprehensive literature review and the exploration of practical and ethical implications are well-taken. We believe these adjustments will signiﬁcantly improve the article’s impact and contribution to the ﬁeld. Thank you for your valuable feedback. The authors respectfully thank the editor and reviewers for their insightful recommendations, which helped to improve this paper.**
